# Compartmentalized replication organelle of flavivirus at the ER and the factors involved

**DOI:** 10.1007/s00018-021-03834-6

**Published:** 2021-04-12

**Authors:** Yali Ci, Lei Shi

**Affiliations:** 1 State Key Laboratory of Medical Molecular Biology, Institute of Basic Medical Sciences, Chinese Academy of Medical Sciences and School of Basic Medicine, Peking Union Medical College, Beijing, 100005 China; 2 Department of Biochemistry and Molecular Biology, Institute of Basic Medical Sciences, Chinese Academy of Medical Sciences and School of Basic Medicine, Peking Union Medical College, Beijing, 100005 China

**Keywords:** Flavivirus, Replication organelle, ER rearrangement, Compartmentalization, Membrane remodeling, Nonstructural proteins, Host factors

## Abstract

Flaviviruses are positive-sense single-stranded RNA viruses that pose a considerable threat to human health. Flaviviruses replicate in compartmentalized replication organelles derived from the host endoplasmic reticulum (ER). The characteristic architecture of flavivirus replication organelles includes invaginated vesicle packets and convoluted membrane structures. Multiple factors, including both viral proteins and host factors, contribute to the biogenesis of the flavivirus replication organelle. Several viral nonstructural (NS) proteins with membrane activity induce ER rearrangement to build replication compartments, and other NS proteins constitute the replication complexes (RC) in the compartments. Host protein and lipid factors facilitate the formation of replication organelles. The lipid membrane, proteins and viral RNA together form the functional compartmentalized replication organelle, in which the flaviviruses efficiently synthesize viral RNA. Here, we reviewed recent advances in understanding the structure and biogenesis of flavivirus replication organelles, and we further discuss the function of virus NS proteins and related host factors as well as their roles in building the replication organelle.

## Introduction

RNA viruses pose a great threat to human beings. Many well-known viruses, such as influenza virus, HIV, dengue virus (DENV), rabies virus, poliovirus and the current global epidemic SARS-CoV-2, are RNA viruses. RNA viruses have a very high mutation rate during their replication, which ensures their fitness for survival and spread in hosts. Flaviviruses are positive-sense single-stranded RNA viruses that have infected humans and caused severe symptoms for several hundred (probably over thousand) years, and comprise 70 members, including yellow fever virus (YFV), Zika virus (ZIKV), DENV and West Nile virus (WNV). Currently, an estimated 400 million DENV infection cases occur annually around the world, and 40% of the world’s population in over 100 counties is at risk of infection [1–3]. During the recent outbreak of ZIKV in America, more than 500,000 cases were reported at the peak of the pandemic in 2016 [4]. Undoubtedly, flaviviruses epidemic have become a great challenge to public health.

Flavivirus is one of the four genera of the *Flaviviridae* family, and the blood-borne hepatitis C virus (HCV) is another related member belong to the genus *Hepacivirus* of *Flaviviridae* family. To avoid confusion, “flavivirus” hereafter in the present review refers to just the members of the Flavivirus genus and does not include HCV. Flaviviruses are also known as arboviruses, because most flaviviruses can be transmitted by arthropod vectors (mosquitoes or ticks). Mosquito-borne flaviviruses can be vertically transmitted between mosquito generations. Meanwhile, mosquito- and tick-borne flaviviruses are horizontally transmitted between mosquitoes/ticks and humans by bites, which makes cross-species transmission of flavivirus possible.

Flaviviruses are enveloped viruses with diameters of approximately 50 nm. The flavivirus genome is a positive-sense single-stranded RNA ~ 11 kb long in length that can be directly translated into a viral polyprotein precursor. The polyprotein precursor is processed by viral protease (NS3 and NS2B) and host proteases (e.g., signal peptidase) and cleaved into three structural proteins and seven nonstructural proteins. Structural proteins (capsid, E and prM/M) assemble viral particles. The capsid proteins associate with viral genomic RNA to form nucleocapsids. E and M proteins are virus envelope proteins involved in flavivirus entry and fusion. Nonstructural proteins participate in virus replication in the cell.

**Fig. 1:**
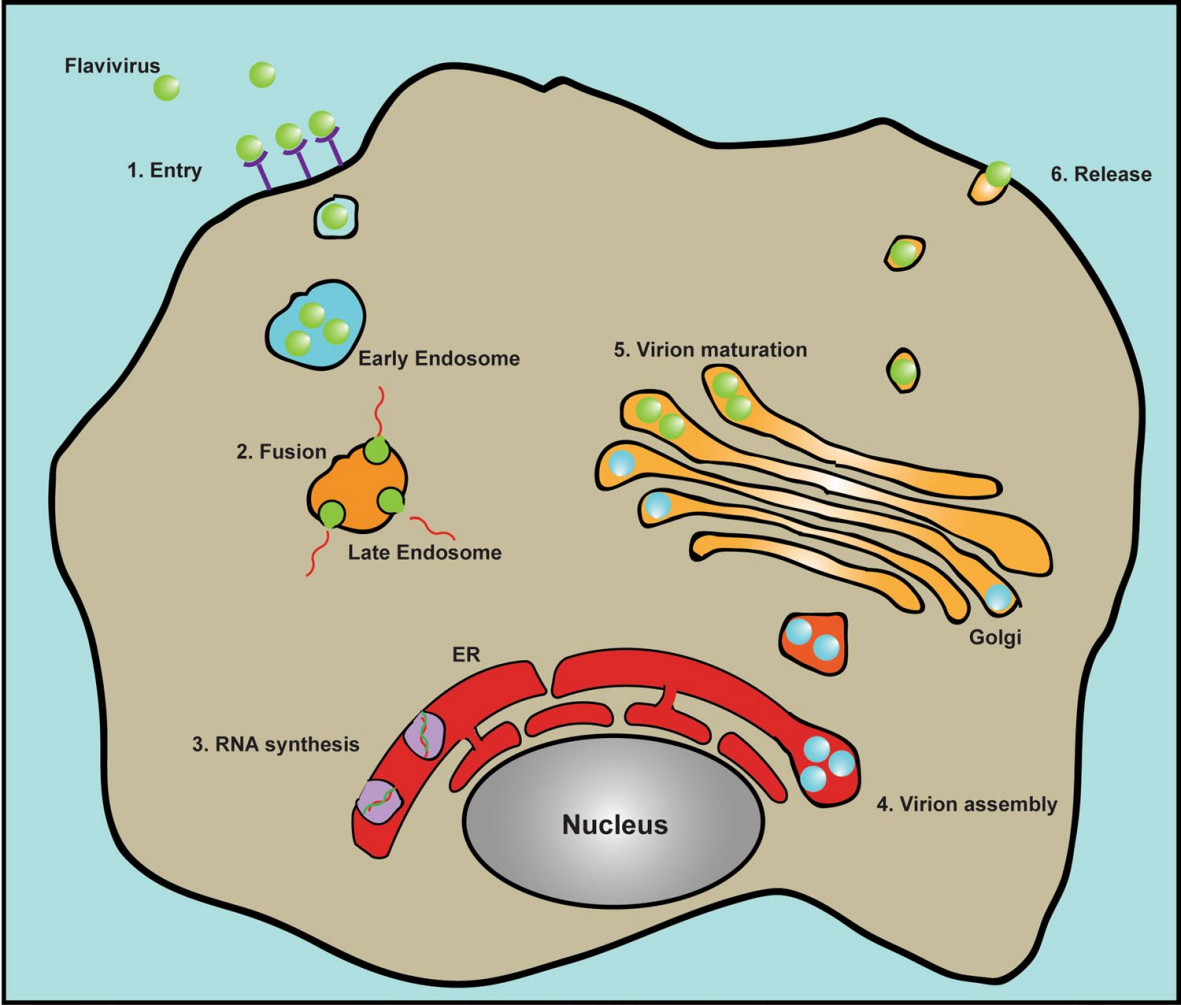
Life cycle of flavivirus. Flavivirus enters cell through endocytosis pathway upon binding to some receptors on cell surface. Viral E protein drives the membrane fusion between viral envelope and endosome membrane to release the nucleocapsid. Flavivirus replication and virion assembly occur at the ER. Immature virion is then transported to the Golgi apparatus and the maturation happens in the trans-Golgi network. Viral particle is finally released outside the cell through exocytosis pathway

Flaviviruses enter the cell through endocytosis. Although various membrane factors are implied to be involved in binding to flaviviral E proteins for virus attachment, specific cellular receptors for flaviviruses entry are still unidentified (for review, see [3, 5]). The E protein is also the fusogen that mediates the fusion between the viral envelop and endosomal membrane to release the virus nucleocapsid. Subsequent replication and virion assembly of flaviviruses occur at the ER. Then, viral particles are transported into the Golgi apparatus for maturation and finally released outside of the cell through the cellular secretary pathway (Fig. 1).

Flavivirus replication in the cell induces remarkable ER rearrangement, described as dilated ER cisternae with multiple invaginated vesicles (Ve), convoluted membranes (CM) and paracrystalline arrays (PC), which is a characteristic structural hallmark of flavivirus infection [6–9]. Compartmentalized and aggregated membranous networks derived from host cellular ER form flavivirus replication platforms. Together with flavivirus RC and viral RNA, they constitute flavivirus replication organelles in the cell. The present review discusses the architecture and biogenesis of flavivirus replication organelles, the factors involved and the underlying mechanism.

## Membranous replication organelle of flavivirus

Flaviviruses are not the only viruses that take advantage of the host intracellular membrane system to replicate. Actually, the replication of almost all positive-strand RNA viruses is associated with cellular membranous organelles (for review, see [10]).

Utilization of the host intracellular membrane system to establish viral replication organelles is essential and has many merits. First, a membranous structure is needed and propitious to the assembly of viral RC. Usually, virus-encoded NS proteins are responsible for replication. To efficiently synthesize virus genome, virus NS proteins assemble into RC to function. Many NS proteins are integral membrane proteins located on cellular organelles. Thus, viral RC assembly on the membranous structure is intrinsically essential. Second, membranous structures, such as single-membrane vesicles (SMVs) or double-membrane vesicles (DMVs), are often formed in the replication of a variety of viruses. These vesicles form a relatively enclosed space and environment. On one hand, such an enclosed structure concentrates the enzyme and substrates for higher reaction efficiency. On the other hand, the compartments also protect the viral genome from recognition, modification and degradation by host cellular immune system and then to evade or delay the host immune response [11–13]. Third, enveloped virus replication sites on the membrane could be spatially close to virion assembly sites, which is convenient for the subsequent virion assembly step. In addition to proximity, some viral replication factors also regulate virion assembly; thus, virus replication and assembly are tightly associated with each other.

As mentioned above, flaviviruses reorganize host cell ER to build viral replication organelles. The ER is a major location for protein synthesis and lipid metabolism in the cell, providing numerous conveniences for viral replication (for review, see [14]). Soon after the translation and processing of flavivirus-encoded proteins, they could directly assemble in situ and initiate viral replication without further translocation to other organelles. The virion assembly of flaviviruses is also completed at the ER, although the assembly site is considered to be close to but different from the replication compartments. Thus, viral protein translation, viral replication and assembly are spatially linked together.

## Architecture of flavivirus replication organelle

Since the 1960s, the flaviviruses replication has been linked to the ER. Electron microscopy (EM) and biochemical studies provided solid evidence. DENV, Japanese encephalitis virus (JEV) and ZIKV were found to replicate around the ER in different cultured mammalian cells (neurons, lymphoblasts and kidney cells) and infected animal tissues [15–20]. DENV also replicated on the ER of mosquito (Aedes albopictus) cells, indicating a conserved replication site between the mammal host and arthropod vector [21]. Membrane fraction analysis from sucrose gradient density ultracentrifugation also demonstrated that DENV viral proteins were associated with membranes and localized on the ER [19]. Structurally, dilated ER cisternae enclosed with vesicles and virions are often described in early EM studies.

**Fig. 2:**
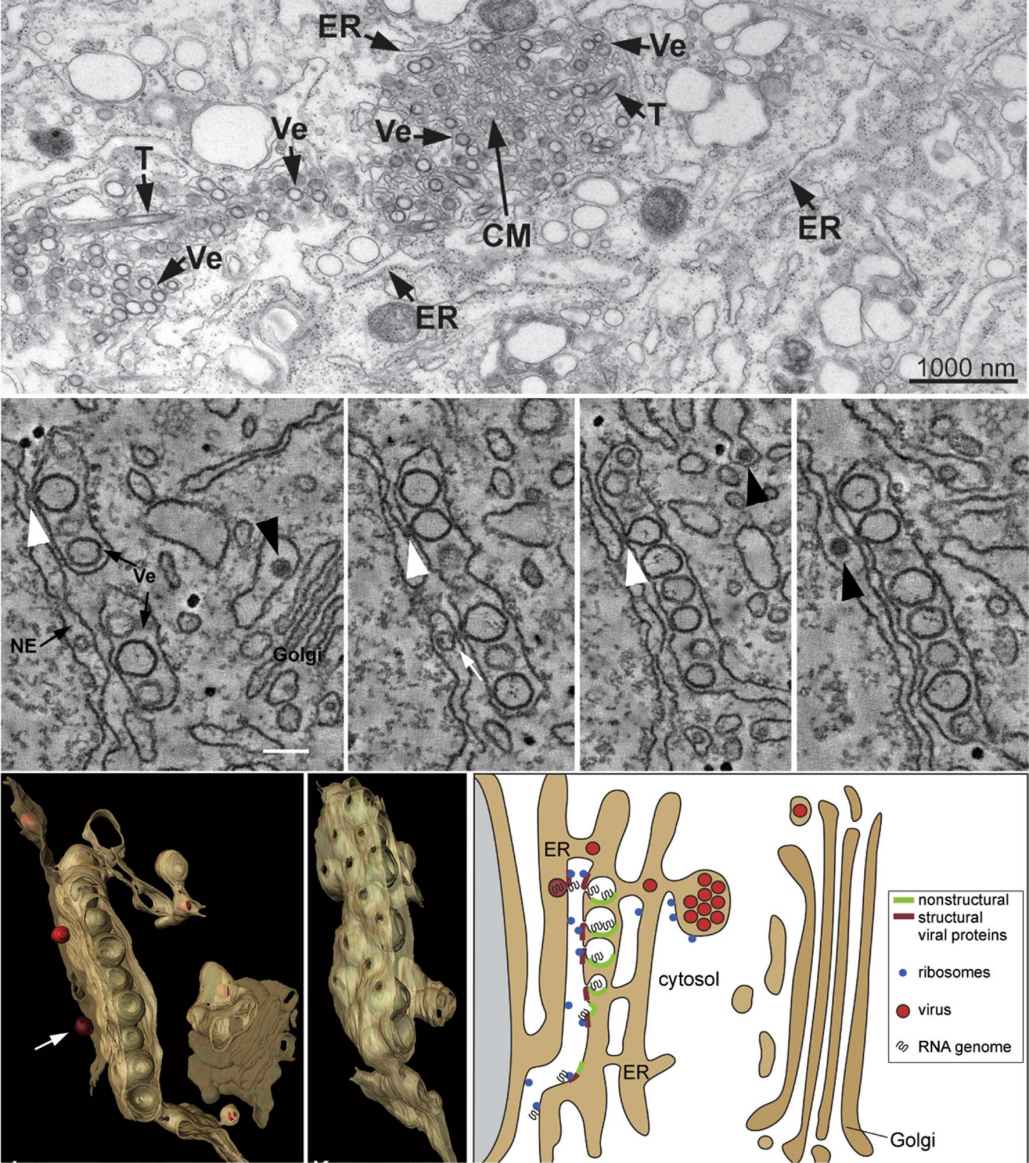
Flavivirus replication organelles derived from the ER. Top panel, TEM image of DENV replication organelles derived from the ER. *T* tubes, *Ve* virus-induced vesicles, *CM* convoluted membranes. Middle panel, continuous slices through an EM tomogram (~ 2 nm thick). White arrowhead, continuity of vesicle and ER membranes; black arrowhead, virus particles. Bottom panel, three-dimensional architecture of flavivirus replication organelles (left) and the model of flavivirus replication, assembly and release (right). These images are reproduced with permission from Ref. [7]

With the development of EM, the fine tuning and three-dimensional structure of flavivirus replication compartments has been successfully resolved (Fig. 2). Using electron tomography, Ralf Bartenschlager group showed that DENV replication compartments are SMVs invaginated toward the dilated ER lumen with a diameter of 80–90 nm, and multiple invaginated vesicles enclosed in the ER lumen form vesicle packets (VPs) [7, 22]. However, these vesicles are not completely sealed structures. A narrow neck (~ 10 nm in diameter) connects the invaginated vesicle with the cytosol to allow for the exchange of materials such as proteins, nucleic acids (newly synthesized viral RNA) and other molecules. CM are also found in the center of aggregated membrane networks. Similar ultrastructure of TBEV, WNV, and ZIKV replication compartments was also recently observed by EM [23–26].

Although HCV belongs to the *Flaviviridae* family and is a different genus, the HCV replication organelle is quite different from flaviviruses [27]. The larger DMV (~ 200 nm diameter on average) in the cytoplasm of HCV-infected hepatoma cells is considered the replication organelle. Considering the different structures and formation mechanisms, we focus on flavivirus replication organelles in the present review.

## Crucial factors involved in flavivirus replication organelle formation

To replicate in the cell efficiently, flaviviruses remodel the ER structure. The remodeled ER structure is quite different from the normal structure. Generally, the ER network comprises flattened sacs (cisternae) connected to the nuclear envelope as well as loose interconnected membrane tubes and sheets. Vesicles containing cargos, such as newly synthesized proteins and lipids, bud from the ER and are transported to other organelles. Many cellular factors in the cytoplasm participate in this ER membrane budding process, including membrane curvature induction (protrusion toward the cytoplasm), maintenance and vesicle shedding. Flavivirus infection induces aggregated and tangled ER networks in the cell. Importantly, a flavivirus-induced vesicular structure invaginated toward the ER lumen is a diametrically opposite process of vesicle budding. Clearly, some factors participate in this ER reorganization process during flavivirus replication, including viral proteins, host proteins and lipids. Considering the rareness of the invaginated vesicular structure at the ER under physiological conditions, the assumption that flavivirus-encoded proteins play dominant roles establishing viral replication organelles is reasonable. Nevertheless, host factors such as proteins and lipids may facilitate the process.

## Flavivirus NS proteins

**Fig. 3:**
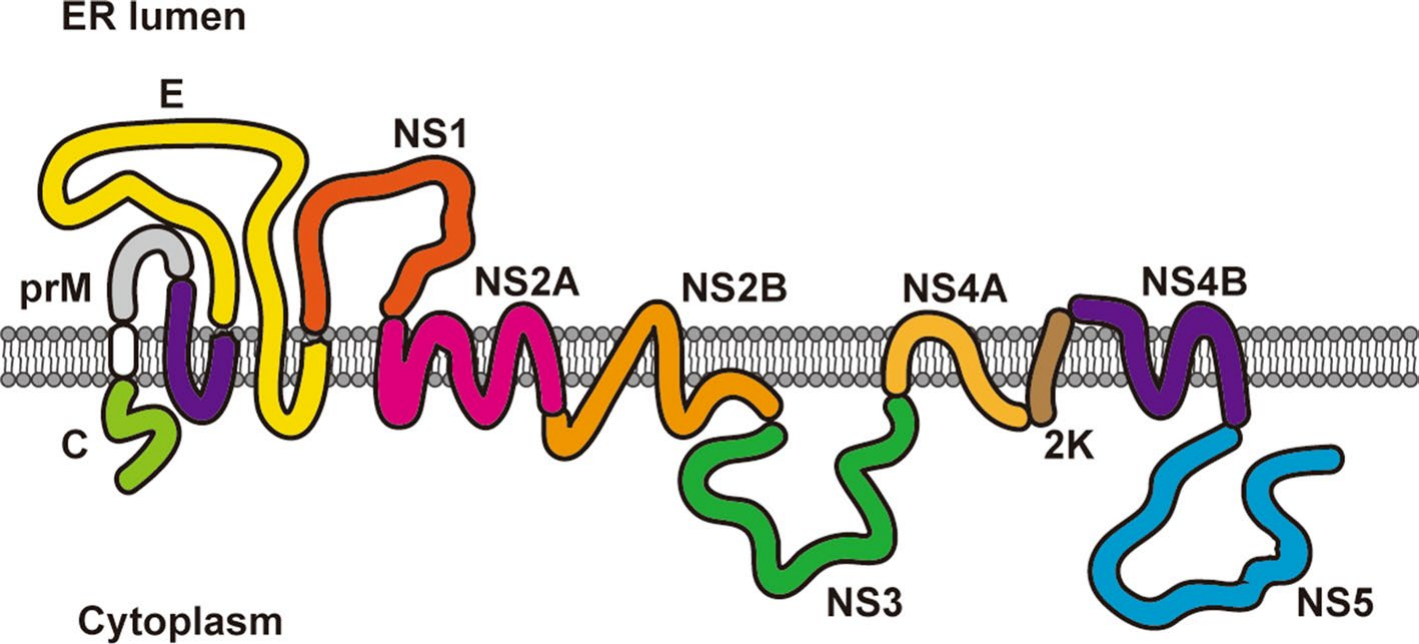
Localization and topology of flavivirus structural and NS proteins. Flavivirus genomic RNA is translated into a multitransmembrane polyprotein precursor on the ER and then cleaved into ten individual proteins by viral and host proteases, including three structural proteins (capsid (C), prM and E) and seven NS proteins (NS1, NS2A, NS2B, NS3, NS4A, NS4B and NS5). NS1 is located in the ER lumen. Capsid, NS3 and NS5 localize in the cytoplasm (some capsid and NS5 are transported into the nucleus). Other viral structural proteins and NS proteins are ER membrane-anchored proteins

Flavivirus genomic RNA encodes ten viral proteins, seven of which are NS proteins (NS1, 2A, 2B, 3, 4A, 4B and 5) that carry out virus replication (Fig. 3). Among the seven NS proteins, NS1 is the only one localized in the ER lumen. NS2A, 2B, 4A and 4B are integral membrane proteins that reside on the ER membrane. NS3 and NS5 are two soluble enzymes with protease, NTPase, helicase and polymerase activity in the cytoplasm. NS3, NS5 and viral RNA need to be recruited to the replication compartments and assembled into functional RC, since they do not possess an ER anchor. Thus, virus replication can be accomplished.

In summary, nonstructural proteins have three functions. First, nonstructural proteins (NS3 and NS5) have enzymatic functions in viral double-stranded RNA (dsRNA) unwinding, RNA synthesis, modification and viral polyprotein processing. Second, nonstructural proteins remodel the host cell ER structure to build viral replication organelles, which is the prerequisite structural basis for replication. Evidence has demonstrated that flavivirus NS proteins are essential and sufficient to establish replication organelles [6, 28, 29]. Third, some nonstructural proteins also play roles as scaffold proteins in virus replication and assembly. ER membrane-anchored NS proteins recruit soluble enzymes (NS3 and NS5 in the cytoplasm) to the ER to form RC, or bring newly synthesized viral RNA and structural proteins together to assemble the virion.

Below, we discuss the role of each flavivirus NS protein in the establishment of replication organelles in detail.

### NS1

Flavivirus NS1 is the first NS protein translated after the structural proteins. After translation, signal peptidase and unidentified host proteases cleave between E/NS1 and NS1/2A, producing the individual NS1 protein. NS1 is the only NS protein localized in the ER lumen, where proteins are glycosylated. The molecular weight of NS1 ranges from 46 to 55 kD depending on glycosylation [30, 31]. Meanwhile, as the ER is the gateway of cellular secretion route, NS1 hitchhike the secretory pathway and is secreted into the extracellular milieu. Thus, it is not surprising that DENV NS1 was first found as soluble complement fixing (SCF) antigen 50 years ago [32, 33].

In addition to substantial studies focusing on secreted NS1, biochemical and structural studies also provide valuable functional information of NS1. Evidence has indicated the association of secreted JEV NS1 with extracellular membranous particles [34]. GPI linkage of DENV NS1 with the membrane was also reported [35]. Structural studies demonstrated that secreted NS1 forms dimers or hexamers associated with lipid membrane or lipoprotein particles [36, 37]. Based on these data, researchers have tried to establish the linkage between NS1 function and the immune response or vascular dysfunction.

However, all the above mentioned studies cannot answer the question “What is the role of NS1 in flavivirus replication?” Neither the induction of the host immune response by secreted NS1 nor the NS1-lipid association seems to have a direct link to flavivirus replication. Flavivirus NS1 has been proved to be essential for virus replication in viral genomic RNA synthesis for two decades [38, 39]. DENV NS1 association with the intracellular membrane, particularly with VPs but not with mature virus particles, and NS1 colocalization with viral dsRNA by cryo-EM provide additional evidence supporting its role in the virus replication complex [39]. Recently, ZIKV NS1 was shown to remodel ER structure in mammalian cells, resembling the flavivirus replication compartments [29]. These data vigorously argue that NS1 is directly involved in the establishment of flavivirus replication organelles and unravel the essential role of NS1 in virus replication mechanistically [29, 40].

**Fig. 4:**
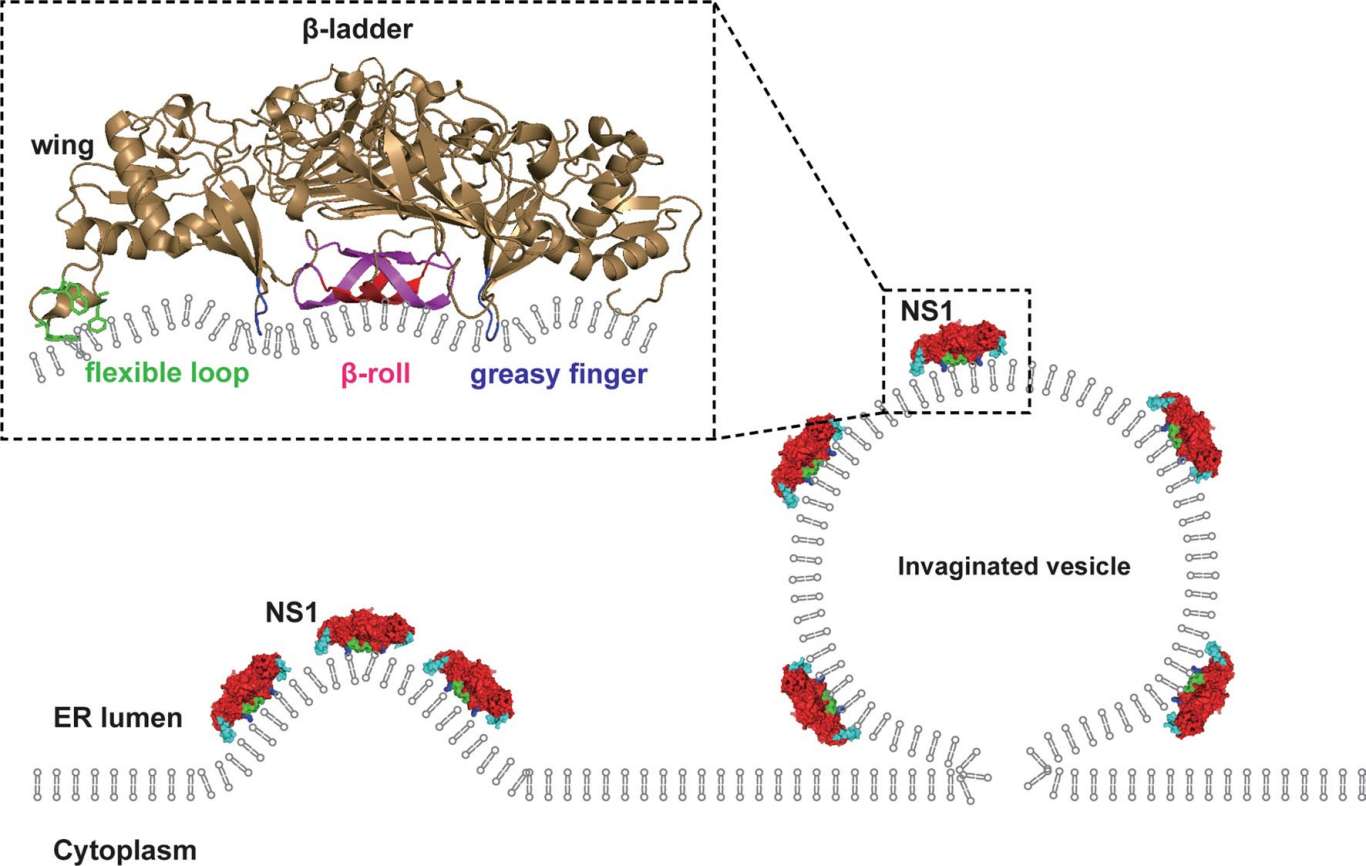
Flavivirus NS1 induces invaginated vesicles at the ER. Several hydrophobic residues in β-roll, greasy finger and wing flexible loop of NS1 can insert into lumenal leaflet of the ER membrane to induce the curvature. Thus, ER lumen located NS1-induced invaginated vesicles at the ER by the mechanism of asymmetrically insertion into the lipid membrane

The essential role of NS1 in viral replication depends on its association with the lipid membrane. Structural studies demonstrated that flavivirus NS1 has three regions facing the lipid membrane: β-roll, greasy finger and wing flexible loop [37, 41, 42]. Several hydrophobic residues in these regions of NS1 could insert into the lumenal leaflet of the ER membrane to introduce membrane curvature toward the ER lumen, resulting in the formation of invaginated vesicles (Fig. 4) [29]. Actually, membrane curvature induction by asymmetric hydrophobic insertion into the lipid bilayer is quite common in the cell. Many cellular factors induce or maintain the vesicular or tubular membrane structure in this way through membrane active domains such as the N-Bar, PH, PX, C1/2 and FYVE domains [43–47].

Flavivirus replication occurs on the cytoplasmic side of the ER, where viral RNA and enzymes (NS3 and NS5) exist. It has long been mystery how NS1 in the ER lumen, sequestered by the ER membrane, plays an essential role in viral RNA synthesis. Some studies have shown that NS1 interacts with other flavivirus NS proteins to participate in virus replication, suggesting that NS1 acts as a cofactor rather than an important player in viral replication. However, this cannot explain why point mutations in the NS1 membrane association region block virus replication. The model by which NS1 induces ER invagination to establish replication organelles converges with several lines of previous studies, including NS1 association with lipids, NS1 ER lumen location around replication compartments and NS1 essential role in viral RNA synthesis.

Flavivirus NS1 is a multifunctional protein involved in various processes (for review, see [48–51]). We do not discuss these processes in detail here due to the relatively weak connection with flavivirus replication organelles.

### NS2A

NS2A (~ 23 kD) is a membrane protein localized at the ER with five membrane span regions [52]. A recent report suggested a single-transmembrane topology for ZIKV NS2A, which differs from that of other flaviviruses [53]. The NS2A N-terminus localizes in the ER lumen, and the C-terminus localizes in the cytosol. Immunofluorescence and immuno-EM demonstrated that NS2A localized around the virus-induced membrane (VP, CM and PC) [54].

Functionally, mutation scanning analysis indicated that DENV and YFV NS2A is involved in both viral RNA synthesis and virion assembly [55–57]. Several reports have identified multiple residues of WNV and ZIKV NS2A affecting viral replication [58–60]. These residues lie throughout the NS2A sequence without obvious clustering patterns, and the underlying mechanisms are not well known. On the other hand, the role of flavivirus NS2A in virion assembly seems more evident [61–63]. In two recent studies, the models in which DENV and ZIKV NS2A may act as an important scaffold protein in virion assembly are proposed: NS2A facilitates virion formation by recruiting c-prM-E structural proteins and viral RNA to the assembly site [64, 65].

A few studies have suggested the membrane activity of DENV NS2A. Dens25, a peptide in the N-terminus of DENV2 NS2A located in the ER lumen, possesses membrane insertion properties in vitro, suggesting its possible involvement in membrane modulation [66, 67]. The data also showed that DENV NS2A exhibited pore forming activity in the bacterial expression system [68]. However, the correlation of these observations with flavivirus replication is ambiguous. Thus, whether NS2A plays a role in replication organelle establishment remains unclear and should be determined.

### NS2B

Flavivirus NS2B (~ 14 kD) is a multitransmembrane protein localized at the ER with four proposed membrane-spanning regions (the C-terminal two helices may be half-buried in the membrane) [69]. Immunofluorescence and cryo-immuno-EM showed that Kunjin virus NS2B colocalized with viral dsRNA around virus-induced membranes [70, 71]. Flavivirus NS2B has a well-known role as an essential cofactor of NS3 protease by increasing its protease activity more than 3000-fold [72–76]. The NS2B-NS3 protease complex structure revealed that the cytosolic region of NS2B wrapped around the NS3 protease domain, stabilizing NS3 protease folding [77–80]. Furthermore, as NS3 has no membrane tethered region, NS2B recruits NS3 to the replication site at the ER [81]. Thus, NS2B plays dual roles in NS3 function: a cofactor of the NS3 protease domain and a recruiter of NS3 to the ER.

A study also showed that NS2B alters membrane structure in vitro. Bacterially expressed JEV and DENV NS2B form oligomers and permeabilize the cell membrane [82, 83]. Interestingly, several mutations in JEV NS2B transmembrane region also attenuated viral RNA synthesis and virion assembly to varying extents independent of NS3 protease activity, one of which was proven to weaken the NS2A-2B interaction [84]. It seems that NS2B has extra functions other than the NS3 cofactor and recruiter in virus replication. However, the role of NS2B, especially in flavivirus-induced membrane structure, is vague and needs to be explored.

### NS4A

NS4A (~ 16 kD) is a small membrane protein with three proposed membrane-spanning regions [85]. Flavivirus NS4A was reported to localize around virus-induced membrane structure in virus-infected cells [54, 86]. The NS4A N-terminus is located in the cytosol after cleavage from NS3, and the C-terminus lies in the ER lumen. The last C-terminal transmembrane region, also known as the 2 K peptide, is the leading signal sequence to introduce the N-terminus of NS4B into the ER lumen.

Evidence has shown that NS4A can induce membrane rearrangement, which may be related to virus replication organelle formation. The 2 K peptide, which is between NS4A and NS4B, probably plays a role in NS4A-induced membrane alteration, although the opposite contribution was reported in DENV and WNV [87, 88]. Moreover, the N-terminal cytoplasmic domain of DENV NS4A contains an amphipathic helix (AH) that may interact with the membrane [89]. AH is a common motif associated with membrane structure comprising hydrophobic and polar residues that form a polar/nonpolar interface. The side chains of hydrophobic residues in the AH insert into the lipid bilayer, and the polar residues interact with the polar region of the membrane. Thus, AH can sense, induce or maintain membrane curvature. DENV NS4A N-terminal AH bound to a highly curved lipid membrane specifically in vitro [90, 91]. It is possible that NS4A interacts with the cytoplasmic leaflet of the ER membrane to sense or modulate the ER structure through its N-terminal AH motif. Molecular dynamics (MD) simulations also suggest that DENV NS4A could induce membrane undulation [92]. In addition, the oligomerization of DENV NS4A mediated by its first TM region is important for virus replication, and mutation in this region reduced NS4A oligomerization and attenuated virus replication [93]. In addition, it is still under discussion whether NS4A interacts with NS1 to facilitate the construction of flavivirus replication organelle [40, 94].

### NS4B

NS4B (~ 27 kD) is an integral membrane protein with three transmembrane regions [95]. NMR experiment also suggests a 5-transmembrane model of NS4B using reconstitute micelles in vitro [96]. The NS4B N-terminus lies in the ER lumen, and the C-terminus is located in the cytoplasm. Its colocalization with NS3 and viral dsRNA on the ER implies its association with the viral membrane-bound replication complex [95]. NS4B participates in DENV replication, as lethal mutations (P104R, K143A) in NS4B have been identified [97]. This could be due to the dimerization of DENV NS4B through the cytosolic loop (129–165 aa) and the C-terminal region (166–248 aa), which is important for replication complex formation [98]. A study also demonstrated that DENV NS4B interacted with NS3 and promoted NS3 helicase activity by dissociating it from single-stranded RNA [99].

For the membrane rearrangement, DENV NS4B possesses several membrane active regions, comprising both the proposed transmembrane region and non-transmembrane regions [100]. Overexpression of WNV NS4B could induce ER-derived foci formation in the cytoplasm by confocal microscopy, suggesting its possible role in virus-induced membrane structure [101]. The role of NS4B in flavivirus-induced ER remodeling needs further study.

### NS3 and NS5

NS3 and NS5 are viral enzymatic proteins carrying out protein processing, dsRNA unwinding, RNA synthesis and modification. NS3 (~ 68 kD) has an N-terminal protease domain and a C-terminal helicase domain to cleave the viral polyprotein and separate the dsRNA intermediate during viral RNA amplification, respectively. NS5 (~ 105 kD) has an N-terminal methyltransferase and a C-terminal RNA-dependent RNA polymerase (RDRP) domain with 5′RNA capping, cap methylation and RNA synthesis activities. These two proteins are soluble proteins without membrane anchors. Thus, for efficient virus replication, NS3 and NS5 should be retained around replication compartments on the ER by other factors. Interestingly, ZIKV NS3 localized on mitochondria when expressed alone, whereas NS2B could recruit NS3 to the ER, where flavivirus replicates [81]. Flavivirus NS5 has both cytoplasmic and nuclear localization, whose nuclear localization may relate to virus replication and the host immune response [102–105]. The factor recruiting NS5 to the replication site at the ER needs to be identified. To date, no evidence indicates that NS3 and NS5 are directly involved in the formation of flavivirus-induced membrane structures [28]. Surprisingly, in addition to its enzymatic function, NS3 seems to play a role in virion assembly. Mutation of several residues in DENV and YFV NS3 does not affect virus replication but abolishes infectious particle assembly, although the underlying mechanism remains to be explored [106, 107].

## Flavivirus compartmentalized replication organelle formation

**Fig. 5:**
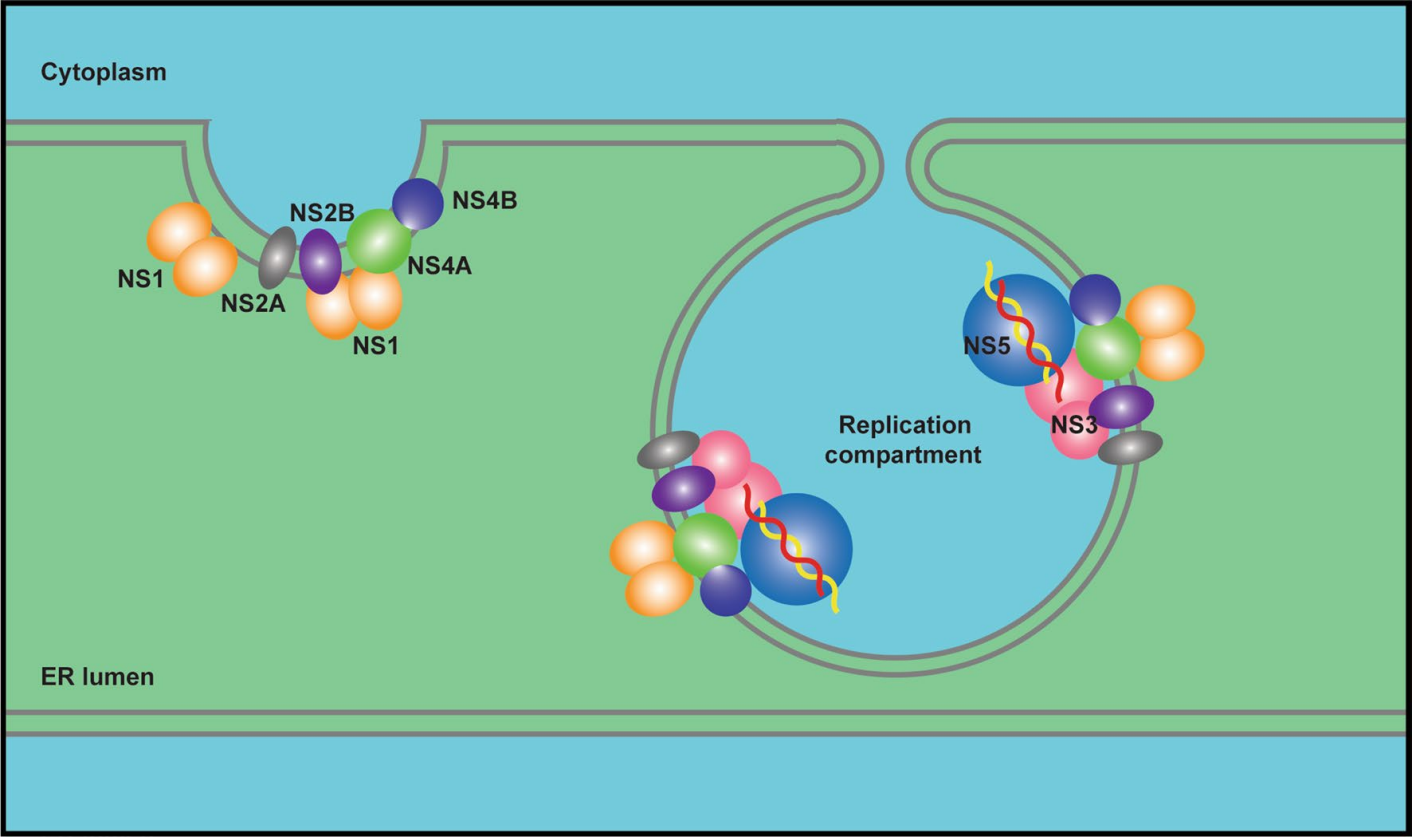
Flavivirus replication organelles at the ER. Membrane active NS proteins induce invaginated replication compartments at the ER, and RC (NS3, NS5 and viral RNA etc.) assemble in such compartments to carry out viral RNA synthesis. Host factors may also contribute to the establishment of flavivirus replication organelles

The formation of flavivirus compartmentalized replication organelle is a complicated process involving membrane remodeling (replication compartment formation on the ER), protein complex assembly (virus RC assembly) in the replication compartment, RC-viral RNA recognition, RNA synthesis and release (Fig. 5). Protein–membrane, protein–protein and protein–RNA interactions together contribute to the formation of flavivirus replication organelles. Thus, the ER membrane, viral nonstructural proteins and viral RNA form a compact and highly ordered structure to accomplish virus replication.

Compartmentalized ER structure is the platform of flavivirus replication. Multiple NS proteins in flaviviruses have been shown to interact with lipid membranes. NS1, as a membrane associated protein, may play a key role in replication compartment formation through hydrophobic insertion into lumenal leaflet of the ER membrane. The other four smaller multitransmembrane NS proteins residing on the ER membrane, NS2A, 2B, 4A and 4B, all possess membrane binding or permeabilizing activity in the model membrane system or bacterial expression system [83]. However, the data from the model system in vitro need to be carefully evaluated to distinguish whether the membrane activities of these NS proteins are actually related to replication compartment formation. Some may be involved in other processes, such as virion assembly or virus-induced cytopathic effects. A more complicated scenario is that a single NS protein may not be sufficient to establish replication compartments alone. Evidence has shown that NS1, NS2B, NS4A and NS4B form homodimers or oligomer to function. Furthermore, there are broad interactions among these NS proteins, including NS1 and NS4, NS2A and NS2B, NS4A and NS4B [40, 108–112]. It is likely that cooperation of multiple NS proteins induces and fine tunes the exquisite architecture of replication organelles.

Once the replication compartments form, RC may assemble in these invaginated vesicles at the ER. Two enzymes, NS3 and NS5, need to be recruited to replication compartments by ER membrane-bound NS proteins. As mentioned above, NS2B recruits NS3, whereas recruiter of NS5 has not been identified. NS3 is a potential factor to recruit NS5 to the flavivirus replication site at the ER as they interact each other. In addition, the cooperation between NS3 (RNA helicase) and NS5 (RNA polymerase) is pivotal and essential for viral RNA synthesis, and they regulate their enzymatic activity reciprocally [113–115]. The stoichiometric proportion of NS3–NS5 in RC would matter for better efficiency. It may not be equal, considering equivalent amounts of NS3 and NS5 are translated and processed from the same viral polyprotein precursor, while a certain amount of NS5 is localized in the nucleus rather than on the ER. Additionally, long-range viral dsRNA (~ 11 kb) unwinding probably requires the cooperation of multiple helicases in view of the limited progressivity of flavivirus NS3 helicase on the nucleic acid strand [113, 116]. Therefore, more NS3 than NS5 may be present in the replication compartments. It is intriguing to clarify the proper ratio and how to maintain this ratio between helicase and polymerase to efficiently synthesize viral RNA. Other NS proteins may also be involved in the RNA synthesis process, facilitating NS3 and NS5 function. For example, DENV and WNV NS4A/B interacted with NS3 and regulated NS3 helicase activity [117–119]. Collectively, precise assembly of RC consisting of certain NS proteins with appropriate proportions in the replication compartment is critical for viral RNA synthesis.

Viral RNA also needs to be recruited to the replication compartment, and newly synthesized positive-strand RNA is released from the compartment. The 5′ and 3′ UTRs of flavivirus RNA form a series of stem-loop structures, which might be recognized by viral proteins for RNA unwinding, synthesis and packaging into viral particles. Moreover, 3′ UTR of DENV genomic RNA is found to play a crucial role in replication compartment biogenesis [120]. Of all NS proteins, NS3 and NS5 are major viral RNA-interacting NS proteins. Apart from NS3 and NS5, NS2A was found to bind to the viral RNA 3′ UTR and coordinate virus packaging. Whether NS2A or other NS proteins interact with viral RNA to facilitate RNA replication is not yet well understood.

## Host cellular factors

Host cellular factors, including proteins and lipids, are important players regulating flavivirus replication organelle assembly. They positively facilitate or negatively attenuate virus replication.

## Protein factors

Lots of host protein factors interact with flavivirus-encoded proteins and viral RNA, and many participate in the host immune response to virus infection (not discussed here). As flaviviruses replicate on the ER, ER proteins are widely reported to participate in and regulate flavivirus replication, including the ER membrane complex (EMC), Hrd1, OST, reticulon, atlastin, etc. [121–126]. Among these proteins, several host protein factors are involved in flavivirus replication by modifying ER structure.

## Reticulon

Reticulon (RTN) is a family of membrane proteins (RTN1–4 in mammals) localized on the ER or plasma membrane (RTN4). ER-associated RTNs are considered as ER-shaping proteins that induce and stabilize curvature of ER tubules by inserting the hairpin-like transmembrane region into the cytoplasmic leaflet of the ER membrane [127]. A study found that RTN 3.1A is recruited to viral replication sites upon WNV, DENV and ZIKV infection [128]. Knockdown of RTN3.1A attenuated flavivirus replication and interfered with virus induced-membrane remodeling. RTN 3.1A specifically interacts with WNV NS4A but not with DENV or ZIKV NS4A, suggesting different regulatory mechanisms.

## Atlastin

Atlastin (ATL) is another protein family that plays a crucial role in ER morphogenesis, particularly in the fusion process of ER tubules. Atlastins are dynamin-related GTPases-mediating homotypic fusion of the ER membrane [129, 130]. Studies suggest atlastin also facilitates flavivirus replication [131, 132]. Silencing of atlastin attenuates ZIKV replication and decreases membrane packets. ATL3 interacts with ZIKV NS2A and NS2B-NS3 [131]. In another study, ATL family proteins played distinct roles in the flaviviruses life cycle. ATL2 knockdown blocked flavivirus replication due to defects in replication organelles. Whereas ATL3 specifically impaired virion maturation and secretion through the ADP-ribosylation factor 4 (Arf4)-related trafficking pathway [132].

## BPI fold containing family B member 3 (BPIFB3)

BPIFB3 is a protein localized to the ER whose function is poorly understood [133]. It belongs to the lipid-binding antimicrobial proteins of the LBP/BPI superfamily based on sequence homology. Silencing of BPIFB3 inhibits flavivirus replication and blocks the formation of replication organelles. EM and biochemical evidence demonstrates that depletion of BPIFB3 promotes reticulophagy (autophagy selectively clears and degrades ER components) [134]. It is not clear whether BPIFB3 directly interacts with viral proteins to regulate flavivirus replication.

## Lipids

Flavivirus infection significantly changes host cell lipid metabolism by adjusting lipid composition and distribution, arguing the importance of lipids [135–138]. Lipids are involved in almost all aspects of the flavivirus life cycle including entry, fusion, replication, assembly and virus crosstalk with the host cell immune response [139–145]. However, only limited studies provided clues to show that specific lipids may be involved in flavivirus replication organelles formation. As the flavivirus replication site, the ER is also an important hub for lipid synthesis and transport. Flaviviruses not only remarkably reorganize ER structure, but they also change the lipid metabolism and composition of the ER. Lipidomic analysis demonstrated that flavivirus infection resulted in pronounced lipid composition changes in the membrane fraction where RC exists in mosquito cells [135]. In another lipidomic study, ZIKV infection induced striking changes of sphingolipids in mammalian cells. Sphingolipid ceramide redistributed to ZIKV replication sites and sensitized cells to ZIKV infection [146]. But the relationship between sphingolipids and ZIKV replication organelles are not clear.

Lipids can contribute to flavivirus replication organelles in various ways. First, lipids intrinsically affect replication organelle structure themselves. Lipids have their own shapes determined by the headgroup and acyl tail. Spontaneous membrane curvature can be induced by the asymmetric distribution of different lipids on the bilayer, depending on lipid type, structures and composition [147]. Cone-shaped ceramide and lysophosphatidylcholine (LPC) naturally possess the ability to induce negative and positive curvature respectively, and both are upregulated in DENV-infected mosquito cells [135]. Lipid composition changes in the ER upon flavivirus infection may assist in the formation of highly curved flavivirus replication organelles [148]. Second, specific lipids on the viral replication membrane may facilitate replication by recruiting related protein factors. Lipids could determine the specificity and increase the affinity of membrane associated protein factors. For example, phosphoinositides, which are a small fraction of cellular lipids, play important roles in signal transduction, intracellular trafficking, organelle structure and homeostasis maintenance. Positive-strand RNA viruses such as enterovirus and HCV regulate PI4P production and distribution, forming PI4P-rich membrane structures essential for virus replication [149]. For flavivirus, evidence showed that the peptide (dens25) of DENV NS2A strongly interacts with the negatively charged lipid membrane, which might be related to membrane rearrangement.

In summarize, although many lipids have been reported to affect flaviviruses replication, and some of them redistribute and concentrated to flaviviruses replication sites [135, 146, 150], no direct evidences were provided to support that these lipids contribute to biogenesis of replication organelles. The role of lipids in the establishment of flavivirus replication organelles should be investigated.

## Conclusion and outlook

Flavivirus-induced compartmentalized replication organelles derived from the ER are a characteristic cellular hallmark, and are the prerequisite and essential structural basis of flavivirus replication. Both viral and host cellular factors are involved, in which viral NS proteins may play dominant roles. The establishment and function of flavivirus replication organelles required multidimensional protein–membrane, protein–protein and protein–RNA interactions as well as orchestration of these protein–membrane, protein–protein and protein–RNA complexes.

Previous studies have extensively explored the function of each flavivirus NS protein, RNA element and host factor in viral replication. However, the functions of individual factors are inevitably affected and modified by other units in the complexes. Thus, such studies could be less comprehensive and inaccurate in consideration of multicomplexes working together rather than monofactor to fulfill the virus replication. To better understand the mechanism of flavivirus replication, integral studies using complexes consisting of multiple factors are necessary. Undoubtedly, the systems and techniques for studying the complexes are more complicated and challenging. Reconstitution of the proteo-liposome system in vitro with more NS proteins in combining with advanced EM and super-resolution microscopy will help to understand the process and underlying mechanism of flavivirus-induced ER membrane remodeling. The live cell imaging and single molecular techniques will help to monitor the real-time dynamics of replication organelles as well as viral RNA synthesis in situ.

## References

[1] Bhatt S, Gething PW, Brady OJ, Messina JP, Farlow AW, Moyes CL, Drake JM, Brownstein JS, Hoen AG, Sankoh O, Myers MF, George DB, Jaenisch T, Wint GR, Simmons CP, Scott TW, Farrar JJ, Hay SI (2013). The global distribution and burden of dengue. Nature.

[2] Wilder-Smith A, Ooi EE, Horstick O, Wills B (2019). Dengue. Lancet.

[3] Pierson TC, Diamond MS (2020). The continued threat of emerging flaviviruses. Nat Microbiol.

[4] Musso D, Ko AI, Baud D (2019). Zika virus infection—after the pandemic. N Engl J Med.

[5] Perera-Lecoin M, Meertens L, Carnec X, Amara A (2013). Flavivirus entry receptors: an update. Viruses.

[6] Mackenzie JM, Khromykh AA, Westaway EG (2001). Stable expression of noncytopathic Kunjin replicons simulates both ultrastructural and biochemical characteristics observed during replication of Kunjin virus. Virology.

[7] Welsch S, Miller S, Romero-Brey I, Merz A, Bleck CK, Walther P, Fuller SD, Antony C, Krijnse-Locker J, Bartenschlager R (2009). Composition and three-dimensional architecture of the dengue virus replication and assembly sites. Cell Host Microbe.

[8] Westaway EG, Ng ML (1980). Replication of flaviviruses: separation of membrane translation sites of Kunjin virus proteins and of cell proteins. Virology.

[9] Ng ML, Pedersen JS, Toh BH, Westaway EG (1983). Immunofluorescent sites in vero cells infected with the flavivirus Kunjin. Arch Virol.

[10] den Boon JA, Ahlquist P (2010). Organelle-like membrane compartmentalization of positive-strand RNA virus replication factories. Annu Rev Microbiol.

[11] Overby AK, Popov VL, Niedrig M, Weber F (2010). Tick-borne encephalitis virus delays interferon induction and hides its double-stranded RNA in intracellular membrane vesicles. J Virol.

[12] Miorin L, Albornoz A, Baba MM, D'Agaro P, Marcello A (2012). Formation of membrane-defined compartments by tick-borne encephalitis virus contributes to the early delay in interferon signaling. Virus Res.

[13] Uchida L, Espada-Murao LA, Takamatsu Y, Okamoto K, Hayasaka D, Yu F, Nabeshima T, Buerano CC, Morita K (2014). The dengue virus conceals double-stranded RNA in the intracellular membrane to escape from an interferon response. Sci Rep.

[14] Ravindran MS, Bagchi P, Cunningham CN, Tsai B (2016). Opportunistic intruders: how viruses orchestrate ER functions to infect cells. Nat Rev Microbiol.

[15] Yasuzumi G, Tsubo I, Sugihara R, Nakai Y (1964). Analysis of the Development of Japanese B Encephalitis (Jbe) Visus. I. Electron microscope studies of microglia infected with Jbe Virus. J Ultrastruct Res.

[16] Bell TM, Field EJ, Narang HK (1971). Zika virus infection of the central nervous system of mice. Arch Gesamte Virusforsch.

[17] Cardiff RD, Russ SB, Brandt WE, Russell PK (1973). Cytological localization of Dengue-2 antigens: an immunological study with ultrastructural correlation. Infect Immun.

[18] Sriurairatna S, Bhamarapravati N, Phalavadhtana O (1973). Dengue virus infection of mice: morphology and morphogenesis of dengue type-2 virus in suckling mouse neurones. Infect Immun.

[19] Stohlman SA, Wisseman CL, Eylar OR, Silverman DJ (1975). Dengue virus-induced modifications of host cell membranes. J Virol.

[20] Sriurairatna S, Bhamarapravati N, Diwan AR, Halstead SB (1978). Ultrastructural studies on dengue virus infection of human lymphoblasts. Infect Immun.

[21] Ko KK, Igarashi A, Fukai K (1979). Electron microscopic observations on Aedes albopictus cells infected with dengue viruses. Arch Virol.

[22] Mackenzie JM, Jones MK, Young PR (1996). Improved membrane preservation of flavivirus-infected cells with cryosectioning. J Virol Methods.

[23] Cortese M, Goellner S, Acosta EG, Neufeldt CJ, Oleksiuk O, Lampe M, Haselmann U, Funaya C, Schieber N, Ronchi P, Schorb M, Pruunsild P, Schwab Y, Chatel-Chaix L, Ruggieri A, Bartenschlager R (2017). Ultrastructural characterization of Zika virus replication factories. Cell Rep.

[24] Miorin L, Romero-Brey I, Maiuri P, Hoppe S, Krijnse-Locker J, Bartenschlager R, Marcello A (2013). Three-dimensional architecture of tick-borne encephalitis virus replication sites and trafficking of the replicated RNA. J Virol.

[25] Junjhon J, Pennington JG, Edwards TJ, Perera R, Lanman J, Kuhn RJ (2014). Ultrastructural characterization and three-dimensional architecture of replication sites in dengue virus-infected mosquito cells. J Virol.

[26] Gillespie LK, Hoenen A, Morgan G, Mackenzie JM (2010). The endoplasmic reticulum provides the membrane platform for biogenesis of the flavivirus replication complex. J Virol.

[27] Romero-Brey I, Merz A, Chiramel A, Lee JY, Chlanda P, Haselman U, Santarella-Mellwig R, Habermann A, Hoppe S, Kallis S, Walther P, Antony C, Krijnse-Locker J, Bartenschlager R (2012). Three-dimensional architecture and biogenesis of membrane structures associated with hepatitis C virus replication. PLoS Pathog.

[28] Yau WL, Nguyen-Dinh V, Larsson E, Lindqvist R, Overby AK, Lundmark R (2019). Model system for the formation of tick-borne encephalitis virus replication compartments without viral RNA replication. J Virol.

[29] Ci Y, Liu ZY, Zhang NN, Niu Y, Yang Y, Xu C, Yang W, Qin CF, Shi L (2020). Zika NS1-induced ER remodeling is essential for viral replication. J Cell Biol.

[30] Muylaert IR, Chambers TJ, Galler R, Rice CM (1996). Mutagenesis of the N-linked glycosylation sites of the yellow fever virus NS1 protein: effects on virus replication and mouse neurovirulence. Virology.

[31] Flamand M, Megret F, Mathieu M, Lepault J, Rey FA, Deubel V (1999). Dengue virus type 1 nonstructural glycoprotein NS1 is secreted from mammalian cells as a soluble hexamer in a glycosylation-dependent fashion. J Virol.

[32] Brandt WE, Chiewslip D, Harris DL, Russell PK (1970). Partial purification and characterization of a dengue virus soluble complement-fixing antigen. J Immunol.

[33] Russell PK, Chiewsilp D, Brandt WE (1970). Immunoprecipitation analysis of soluble complement-fixing antigens of dengue viruses. J Immunol.

[34] Mason PW (1989). Maturation of Japanese encephalitis virus glycoproteins produced by infected mammalian and mosquito cells. Virology.

[35] Jacobs MG, Robinson PJ, Bletchly C, Mackenzie JM, Young PR (2000). Dengue virus nonstructural protein 1 is expressed in a glycosyl-phosphatidylinositol-linked form that is capable of signal transduction. FASEB J.

[36] Gutsche I, Coulibaly F, Voss JE, Salmon J, d'Alayer J, Ermonval M, Larquet E, Charneau P, Krey T, Megret F, Guittet E, Rey FA, Flamand M (2011). Secreted dengue virus nonstructural protein NS1 is an atypical barrel-shaped high-density lipoprotein. Proc Natl Acad Sci U S A.

[37] Akey DL, Brown WC, Dutta S, Konwerski J, Jose J, Jurkiw TJ, DelProposto J, Ogata CM, Skiniotis G, Kuhn RJ, Smith JL (2014). Flavivirus NS1 structures reveal surfaces for associations with membranes and the immune system. Science.

[38] Lindenbach BD, Rice CM (1997). trans-Complementation of yellow fever virus NS1 reveals a role in early RNA replication. J Virol.

[39] Mackenzie JM, Jones MK, Young PR (1996). Immunolocalization of the dengue virus nonstructural glycoprotein NS1 suggests a role in viral RNA replication. Virology.

[40] Plaszczyca A, Scaturro P, Neufeldt CJ, Cortese M, Cerikan B, Ferla S, Brancale A, Pichlmair A, Bartenschlager R (2019). A novel interaction between dengue virus nonstructural protein 1 and the NS4A-2K-4B precursor is required for viral RNA replication but not for formation of the membranous replication organelle. PLoS Pathog.

[41] Brown WC, Akey DL, Konwerski JR, Tarrasch JT, Skiniotis G, Kuhn RJ, Smith JL (2016). Extended surface for membrane association in Zika virus NS1 structure. Nat Struct Mol Biol.

[42] Xu X, Song H, Qi J, Liu Y, Wang H, Su C, Shi Y, Gao GF (2016). Contribution of intertwined loop to membrane association revealed by Zika virus full-length NS1 structure. EMBO J.

[43] Dumas JJ, Merithew E, Sudharshan E, Rajamani D, Hayes S, Lawe D, Corvera S, Lambright DG (2001). Multivalent endosome targeting by homodimeric EEA1. Mol Cell.

[44] Kanai F, Liu H, Field SJ, Akbary H, Matsuo T, Brown GE, Cantley LC, Yaffe MB (2001). The PX domains of p47phox and p40phox bind to lipid products of PI(3)K. Nat Cell Biol.

[45] Harlan JE, Hajduk PJ, Yoon HS, Fesik SW (1994). Pleckstrin homology domains bind to phosphatidylinositol-4,5-bisphosphate. Nature.

[46] Salzer U, Kostan J, Djinovic-Carugo K (2017). Deciphering the BAR code of membrane modulators. Cell Mol Life Sci.

[47] Cho W, Stahelin RV (2005). Membrane-protein interactions in cell signaling and membrane trafficking. Annu Rev Biophys Biomol Struct.

[48] Shi Y, Dai L, Song H, Gao GF (2018). Structures of Zika virus E & NS1: relations with virus infection and host immune responses. Adv Exp Med Biol.

[49] Glasner DR, Puerta-Guardo H, Beatty PR, Harris E (2018). The Good, the bad, and the shocking: the multiple roles of Dengue virus nonstructural protein 1 in protection and pathogenesis. Annu Rev Virol.

[50] Rastogi M, Sharma N, Singh SK (2016). Flavivirus NS1: a multifaceted enigmatic viral protein. Virol J.

[51] Watterson D, Modhiran N, Young PR (2016). The many faces of the flavivirus NS1 protein offer a multitude of options for inhibitor design. Antiviral Res.

[52] Xie X, Gayen S, Kang C, Yuan Z, Shi PY (2013). Membrane topology and function of dengue virus NS2A protein. J Virol.

[53] Zhang X, Xie X, Zou J, Xia H, Shan C, Chen X, Shi PY (2019). Genetic and biochemical characterizations of Zika virus NS2A protein. Emerg Microbes Infect.

[54] Mackenzie JM, Khromykh AA, Jones MK, Westaway EG (1998). Subcellular localization and some biochemical properties of the flavivirus Kunjin nonstructural proteins NS2A and NS4A. Virology.

[55] Xie X, Zou J, Puttikhunt C, Yuan Z, Shi PY (2015). Two distinct sets of NS2A molecules are responsible for dengue virus RNA synthesis and virion assembly. J Virol.

[56] Vossmann S, Wieseler J, Kerber R, Kummerer BM (2015). A basic cluster in the N terminus of yellow fever virus NS2A contributes to infectious particle production. J Virol.

[57] Wu RH, Tsai MH, Chao DY, Yueh A (2015). Scanning mutagenesis studies reveal a potential intramolecular interaction within the C-terminal half of dengue virus NS2A involved in viral RNA replication and virus assembly and secretion. J Virol.

[58] Rossi SL, Fayzulin R, Dewsbury N, Bourne N, Mason PW (2007). Mutations in West Nile virus nonstructural proteins that facilitate replicon persistence in vitro attenuate virus replication in vitro and in vivo. Virology.

[59] Yoshii K, Igarashi M, Ito K, Kariwa H, Holbrook MR, Takashima I (2011). Construction of an infectious cDNA clone for Omsk hemorrhagic fever virus, and characterization of mutations in NS2A and NS5. Virus Res.

[60] Marquez-Jurado S, Nogales A, Avila-Perez G, Iborra FJ, Martinez-Sobrido L, Almazan F (2018). An Alanine-to-valine substitution in the residue 175 of Zika virus NS2A protein affects viral RNA synthesis and attenuates the virus in vivo. Viruses.

[61] Kummerer BM, Rice CM (2002). Mutations in the yellow fever virus nonstructural protein NS2A selectively block production of infectious particles. J Virol.

[62] Liu WJ, Chen HB, Khromykh AA (2003). Molecular and functional analyses of Kunjin virus infectious cDNA clones demonstrate the essential roles for NS2A in virus assembly and for a nonconservative residue in NS3 in RNA replication. J Virol.

[63] Leung JY, Pijlman GP, Kondratieva N, Hyde J, Mackenzie JM, Khromykh AA (2008). Role of nonstructural protein NS2A in flavivirus assembly. J Virol.

[64] Zhang X, Xie X, Xia H, Zou J, Huang L, Popov VL, Chen X, Shi PY (2019). Zika Virus NS2A-mediated virion assembly. MBio.

[65] Xie X, Zou J, Zhang X, Zhou Y, Routh AL, Kang C, Popov VL, Chen X, Wang QY, Dong H, Shi PY (2019). Dengue NS2A protein orchestrates virus assembly. Cell Host Microbe.

[66] Nemesio H, Villalain J (2014). Membrane interacting regions of Dengue virus NS2A protein. J Phys Chem B.

[67] Fajardo-Sanchez E, Galiano V, Villalain J (2017). Spontaneous membrane insertion of a dengue virus NS2A peptide. Arch Biochem Biophys.

[68] Shrivastava G, Garcia-Cordero J, Leon-Juarez M, Oza G, Tapia-Ramirez J, Villegas-Sepulveda N, Cedillo-Barron L (2017). NS2A comprises a putative viroporin of Dengue virus 2. Virulence.

[69] Li Y, Li Q, Wong YL, Liew LS, Kang C (2015) Membrane topology of NS2B of dengue virus revealed by NMR spectroscopy. Biochim Biophys Acta 1848 (10 Pt A):2244–2252. 10.1016/j.bbamem.2015.06.010 10.1016/j.bbamem.2015.06.010 26072288

[70] Westaway EG, Mackenzie JM, Kenney MT, Jones MK, Khromykh AA (1997). Ultrastructure of Kunjin virus-infected cells: colocalization of NS1 and NS3 with double-stranded RNA, and of NS2B with NS3, in virus-induced membrane structures. J Virol.

[71] Chu PW, Westaway EG (1992). Molecular and ultrastructural analysis of heavy membrane fractions associated with the replication of Kunjin virus RNA. Arch Virol.

[72] Falgout B, Pethel M, Zhang YM, Lai CJ (1991). Both nonstructural proteins NS2B and NS3 are required for the proteolytic processing of dengue virus nonstructural proteins. J Virol.

[73] Falgout B, Miller RH, Lai CJ (1993). Deletion analysis of dengue virus type 4 nonstructural protein NS2B: identification of a domain required for NS2B-NS3 protease activity. J Virol.

[74] Chambers TJ, Grakoui A, Rice CM (1991). Processing of the yellow fever virus nonstructural polyprotein: a catalytically active NS3 proteinase domain and NS2B are required for cleavages at dibasic sites. J Virol.

[75] Chambers TJ, Nestorowicz A, Amberg SM, Rice CM (1993). Mutagenesis of the yellow fever virus NS2B protein: effects on proteolytic processing, NS2B-NS3 complex formation, and viral replication. J Virol.

[76] Yusof R, Clum S, Wetzel M, Murthy HM, Padmanabhan R (2000). Purified NS2B/NS3 serine protease of dengue virus type 2 exhibits cofactor NS2B dependence for cleavage of substrates with dibasic amino acids in vitro. J Biol Chem.

[77] Erbel P, Schiering N, D'Arcy A, Renatus M, Kroemer M, Lim SP, Yin Z, Keller TH, Vasudevan SG, Hommel U (2006). Structural basis for the activation of flaviviral NS3 proteases from dengue and West Nile virus. Nat Struct Mol Biol.

[78] Lei J, Hansen G, Nitsche C, Klein CD, Zhang L, Hilgenfeld R (2016). Crystal structure of Zika virus NS2B-NS3 protease in complex with a boronate inhibitor. Science.

[79] Phoo WW, Li Y, Zhang Z, Lee MY, Loh YR, Tan YB, Ng EY, Lescar J, Kang C, Luo D (2016). Structure of the NS2B-NS3 protease from Zika virus after self-cleavage. Nat Commun.

[80] Zhang Z, Li Y, Loh YR, Phoo WW, Hung AW, Kang C, Luo D (2016). Crystal structure of unlinked NS2B-NS3 protease from Zika virus. Science.

[81] Xing H, Xu S, Jia F, Yang Y, Xu C, Qin C, Shi L (2020). Zika NS2B is a crucial factor recruiting NS3 to the ER and activating its protease activity. Virus Res.

[82] Leon-Juarez M, Martinez-Castillo M, Shrivastava G, Garcia-Cordero J, Villegas-Sepulveda N, Mondragon-Castelan M, Mondragon-Flores R, Cedillo-Barron L (2016). Recombinant Dengue virus protein NS2B alters membrane permeability in different membrane models. Virol J.

[83] Chang YS, Liao CL, Tsao CH, Chen MC, Liu CI, Chen LK, Lin YL (1999). Membrane permeabilization by small hydrophobic nonstructural proteins of Japanese encephalitis virus. J Virol.

[84] Li XD, Deng CL, Ye HQ, Zhang HL, Zhang QY, Chen DD, Zhang PT, Shi PY, Yuan ZM, Zhang B (2016). Transmembrane domains of NS2B contribute to both Viral RNA replication and particle formation in Japanese Encephalitis virus. J Virol.

[85] Li Y, Lee MY, Loh YR (1860). Kang C (2018) Secondary structure and membrane topology of dengue virus NS4A protein in micelles. Biochim Biophys Acta Biomembr.

[86] Zhang L, Mohan PM, Padmanabhan R (1992). Processing and localization of Dengue virus type 2 polyprotein precursor NS3-NS4A-NS4B-NS5. J Virol.

[87] Miller S, Kastner S, Krijnse-Locker J, Buhler S, Bartenschlager R (2007). The non-structural protein 4A of dengue virus is an integral membrane protein inducing membrane alterations in a 2K-regulated manner. J Biol Chem.

[88] Roosendaal J, Westaway EG, Khromykh A, Mackenzie JM (2006). Regulated cleavages at the West Nile virus NS4A-2K-NS4B junctions play a major role in rearranging cytoplasmic membranes and Golgi trafficking of the NS4A protein. J Virol.

[89] Stern O, Hung YF, Valdau O, Yaffe Y, Harris E, Hoffmann S, Willbold D, Sklan EH (2013). An N-terminal amphipathic helix in dengue virus nonstructural protein 4A mediates oligomerization and is essential for replication. J Virol.

[90] Hung YF, Schwarten M, Schunke S, Thiagarajan-Rosenkranz P, Hoffmann S, Sklan EH, Willbold D, Koenig BW (2015). Dengue virus NS4A cytoplasmic domain binding to liposomes is sensitive to membrane curvature. Biochim Biophys Acta.

[91] Hung YF, Schwarten M, Hoffmann S, Willbold D, Sklan EH, Koenig B (2015). Amino terminal region of Dengue virus NS4A cytosolic domain binds to highly curved liposomes. Viruses.

[92] Lin MH, Hsu HJ, Bartenschlager R, Fischer WB (2014). Membrane undulation induced by NS4A of Dengue virus: a molecular dynamics simulation study. J Biomol Struct Dyn.

[93] Lee CM, Xie X, Zou J, Li SH, Lee MY, Dong H, Qin CF, Kang C, Shi PY (2015). Determinants of Dengue virus NS4A protein oligomerization. J Virol.

[94] Lindenbach BD, Rice CM (1999). Genetic interaction of flavivirus nonstructural proteins NS1 and NS4A as a determinant of replicase function. J Virol.

[95] Miller S, Sparacio S, Bartenschlager R (2006). Subcellular localization and membrane topology of the Dengue virus type 2 Non-structural protein 4B. J Biol Chem.

[96] Li Y, Wong YL, Lee MY, Li Q, Wang QY, Lescar J, Shi PY, Kang C (2016). Secondary structure and membrane topology of the full-length Dengue virus NS4B in micelles. Angew Chem Int Ed Engl.

[97] Xie X, Wang QY, Xu HY, Qing M, Kramer L, Yuan Z, Shi PY (2011). Inhibition of dengue virus by targeting viral NS4B protein. J Virol.

[98] Zou J, Xie X, le Lee T, Chandrasekaran R, Reynaud A, Yap L, Wang QY, Dong H, Kang C, Yuan Z, Lescar J, Shi PY (2014). Dimerization of flavivirus NS4B protein. J Virol.

[99] Umareddy I, Chao A, Sampath A, Gu F, Vasudevan SG (2006). Dengue virus NS4B interacts with NS3 and dissociates it from single-stranded RNA. J Gen Virol.

[100] Nemesio H, Palomares-Jerez F (1818). Villalain J (2012) NS4A and NS4B proteins from dengue virus: membranotropic regions. Biochim Biophys Acta.

[101] Kaufusi PH, Kelley JF, Yanagihara R, Nerurkar VR (2014). Induction of endoplasmic reticulum-derived replication-competent membrane structures by West Nile virus non-structural protein 4B. PLoS ONE.

[102] Ji W, Luo G (2020). Zika virus NS5 nuclear accumulation is protective of protein degradation and is required for viral RNA replication. Virology.

[103] Zhao Z, Tao M, Han W, Fan Z, Imran M, Cao S, Ye J (2019). Nuclear localization of Zika virus NS5 contributes to suppression of type I interferon production and response. J Gen Virol.

[104] Kumar A, Buhler S, Selisko B, Davidson A, Mulder K, Canard B, Miller S, Bartenschlager R (2013). Nuclear localization of dengue virus nonstructural protein 5 does not strictly correlate with efficient viral RNA replication and inhibition of type I interferon signaling. J Virol.

[105] Lopez-Denman AJ, Russo A, Wagstaff KM, White PA, Jans DA, Mackenzie JM (2018). Nucleocytoplasmic shuttling of the West Nile virus RNA-dependent RNA polymerase NS5 is critical to infection. Cell Microbiol.

[106] Gebhard LG, Iglesias NG, Byk LA, Filomatori CV, De Maio FA, Gamarnik AV (2016). A Proline-Rich N-Terminal region of the Dengue Virus NS3 is crucial for infectious particle production. J Virol.

[107] Patkar CG, Kuhn RJ (2008). Yellow Fever virus NS3 plays an essential role in virus assembly independent of its known enzymatic functions. J Virol.

[108] Yu L, Takeda K, Markoff L (2013). Protein-protein interactions among West Nile non-structural proteins and transmembrane complex formation in mammalian cells. Virology.

[109] Youn S, Li T, McCune BT, Edeling MA, Fremont DH, Cristea IM, Diamond MS (2012). Evidence for a genetic and physical interaction between nonstructural proteins NS1 and NS4B that modulates replication of West Nile virus. J Virol.

[110] Wu RH, Tsai MH, Tsai KN, Tian JN, Wu JS, Wu SY, Chern JH, Chen CH, Yueh A (2017). Mutagenesis of dengue virus protein NS2A revealed a novel domain responsible for virus-induced cytopathic effect and interactions between NS2A and NS2B transmembrane segments. J Virol.

[111] Zou J, Xie X, Wang QY, Dong H, Lee MY, Kang C, Yuan Z, Shi PY (2015). Characterization of dengue virus NS4A and NS4B protein interaction. J Virol.

[112] Li XD, Ye HQ, Deng CL, Liu SQ, Zhang HL, Shang BD, Shi PY, Yuan ZM, Zhang B (2015). Genetic interaction between NS4A and NS4B for replication of Japanese encephalitis virus. J Gen Virol.

[113] Xu S, Ci Y, Wang L, Yang Y, Zhang L, Xu C, Qin C, Shi L (2019). Zika virus NS3 is a canonical RNA helicase stimulated by NS5 RNA polymerase. Nucleic Acids Res.

[114] Zhang C, Cai Z, Kim YC, Kumar R, Yuan F, Shi PY, Kao C, Luo G (2005). Stimulation of hepatitis C virus (HCV) nonstructural protein 3 (NS3) helicase activity by the NS3 protease domain and by HCV RNA-dependent RNA polymerase. J Virol.

[115] Zou G, Chen YL, Dong H, Lim CC, Yap LJ, Yau YH, Shochat SG, Lescar J, Shi PY (2011). Functional analysis of two cavities in flavivirus NS5 polymerase. J Biol Chem.

[116] Pang PS, Jankowsky E, Planet PJ, Pyle AM (2002). The hepatitis C viral NS3 protein is a processive DNA helicase with cofactor enhanced RNA unwinding. EMBO J.

[117] Zou J, le Lee T, Wang QY, Xie X, Lu S, Yau YH, Yuan Z, Geifman Shochat S, Kang C, Lescar J, Shi PY (2015). Mapping the Interactions between the NS4B and NS3 proteins of dengue virus. J Virol.

[118] Chatel-Chaix L, Fischl W, Scaturro P, Cortese M, Kallis S, Bartenschlager M, Fischer B, Bartenschlager R (2015). A combined genetic-proteomic approach identifies residues within Dengue virus NS4B critical for interaction with NS3 and viral replication. J Virol.

[119] Shiryaev SA, Chernov AV, Aleshin AE, Shiryaeva TN, Strongin AY (2009). NS4A regulates the ATPase activity of the NS3 helicase: a novel cofactor role of the non-structural protein NS4A from West Nile virus. J Gen Virol.

[120] Cerikan B, Goellner S, Neufeldt CJ, Haselmann U, Mulder K, Chatel-Chaix L, Cortese M, Bartenschlager R (2020). A Non-replicative role of the 3' terminal sequence of the Dengue virus genome in membranous replication organelle formation. Cell Rep.

[121] Zhang R, Miner JJ, Gorman MJ, Rausch K, Ramage H, White JP, Zuiani A, Zhang P, Fernandez E, Zhang Q, Dowd KA, Pierson TC, Cherry S, Diamond MS (2016). A CRISPR screen defines a signal peptide processing pathway required by flaviviruses. Nature.

[122] Marceau CD, Puschnik AS, Majzoub K, Ooi YS, Brewer SM, Fuchs G, Swaminathan K, Mata MA, Elias JE, Sarnow P, Carette JE (2016). Genetic dissection of Flaviviridae host factors through genome-scale CRISPR screens. Nature.

[123] Savidis G, McDougall WM, Meraner P, Perreira JM, Portmann JM, Trincucci G, John SP, Aker AM, Renzette N, Robbins DR, Guo Z, Green S, Kowalik TF, Brass AL (2016). Identification of Zika virus and Dengue virus dependency factors using functional genomics. Cell Rep.

[124] Ma H, Dang Y, Wu Y, Jia G, Anaya E, Zhang J, Abraham S, Choi JG, Shi G, Qi L, Manjunath N, Wu H (2015). A CRISPR-based screen identifies genes essential for West-Nile-Virus-induced cell death. Cell Rep.

[125] Lin DL, Cherepanova NA, Bozzacco L, MacDonald MR, Gilmore R, Tai AW (2017). Dengue virus hijacks a noncanonical oxidoreductase function of a cellular oligosaccharyltransferase complex. MBio.

[126] Hafirassou ML, Meertens L, Umana-Diaz C, Labeau A, Dejarnac O, Bonnet-Madin L, Kummerer BM, Delaugerre C, Roingeard P, Vidalain PO, Amara A (2017). A global interactome map of the Dengue virus NS1 identifies virus restriction and dependency host factors. Cell Rep.

[127] Di Sano F, Bernardoni P, Piacentini M (2012). The reticulons: guardians of the structure and function of the endoplasmic reticulum. Exp Cell Res.

[128] Aktepe TE, Liebscher S, Prier JE, Simmons CP, Mackenzie JM (2017). The host protein Reticulon 3.1A is utilized by flaviviruses to facilitate membrane remodelling. Cell Rep.

[129] Hu J, Prinz WA, Rapoport TA (2011). Weaving the web of ER tubules. Cell.

[130] Goyal U, Blackstone C (2013). Untangling the web: mechanisms underlying ER network formation. Biochim Biophys Acta.

[131] Monel B, Rajah MM, Hafirassou ML, Sid Ahmed S, Burlaud-Gaillard J, Zhu PP, Nevers Q, Buchrieser J, Porrot F, Meunier C, Amraoui S, Chazal M, Salles A, Jouvenet N, Roingeard P, Blackstone C, Amara A, Schwartz O (2019). Atlastin endoplasmic reticulum-shaping proteins facilitate Zika virus replication. J Virol.

[132] Neufeldt CJ, Cortese M, Scaturro P, Cerikan B, Wideman JG, Tabata K, Moraes T, Oleksiuk O, Pichlmair A, Bartenschlager R (2019). ER-shaping atlastin proteins act as central hubs to promote flavivirus replication and virion assembly. Nat Microbiol.

[133] Delorme-Axford E, Morosky S, Bomberger J, Stolz DB, Jackson WT, Coyne CB (2014). BPIFB3 regulates autophagy and coxsackievirus B replication through a noncanonical pathway independent of the core initiation machinery. MBio.

[134] Evans AS, Lennemann NJ, Coyne CB (2020). BPIFB3 regulates endoplasmic reticulum morphology to facilitate flavivirus replication. J Virol.

[135] Perera R, Riley C, Isaac G, Hopf-Jannasch AS, Moore RJ, Weitz KW, Pasa-Tolic L, Metz TO, Adamec J, Kuhn RJ (2012). Dengue virus infection perturbs lipid homeostasis in infected mosquito cells. PLoS Pathog.

[136] Heaton NS, Randall G (2010). Dengue virus-induced autophagy regulates lipid metabolism. Cell Host Microbe.

[137] Zhou J, Chi X, Cheng M, Huang X, Liu X, Fan J, Xu H, Lin T, Shi L, Qin C, Yang W (2019). Zika virus degrades the omega-3 fatty acid transporter Mfsd2a in brain microvascular endothelial cells and impairs lipid homeostasis. Sci Adv.

[138] Chotiwan N, Andre BG, Sanchez-Vargas I, Islam MN, Grabowski JM, Hopf-Jannasch A, Gough E, Nakayasu E, Blair CD, Belisle JT, Hill CA, Kuhn RJ, Perera R (2018). Dynamic remodeling of lipids coincides with dengue virus replication in the midgut of Aedes aegypti mosquitoes. PLoS Pathog.

[139] Martin-Acebes MA, Vazquez-Calvo A, Saiz JC (2016). Lipids and flaviviruses, present and future perspectives for the control of dengue, Zika, and West Nile viruses. Prog Lipid Res.

[140] Lee CJ, Lin HR, Liao CL, Lin YL (2008). Cholesterol effectively blocks entry of flavivirus. J Virol.

[141] Martin-Acebes MA, Merino-Ramos T, Blazquez AB, Casas J, Escribano-Romero E, Sobrino F, Saiz JC (2014). The composition of West Nile virus lipid envelope unveils a role of sphingolipid metabolism in flavivirus biogenesis. J Virol.

[142] Heaton NS, Perera R, Berger KL, Khadka S, Lacount DJ, Kuhn RJ, Randall G (2010). Dengue virus nonstructural protein 3 redistributes fatty acid synthase to sites of viral replication and increases cellular fatty acid synthesis. Proc Natl Acad Sci U S A.

[143] Samsa MM, Mondotte JA, Iglesias NG, Assuncao-Miranda I, Barbosa-Lima G, Da Poian AT, Bozza PT, Gamarnik AV (2009). Dengue virus capsid protein usurps lipid droplets for viral particle formation. PLoS Pathog.

[144] Zaitseva E, Yang ST, Melikov K, Pourmal S, Chernomordik LV (2010). Dengue virus ensures its fusion in late endosomes using compartment-specific lipids. PLoS Pathog.

[145] Zhang J, Lan Y, Li MY, Lamers MM, Fusade-Boyer M, Klemm E, Thiele C, Ashour J, Sanyal S (2018) Flaviviruses exploit the lipid droplet protein AUP1 to trigger lipophagy and drive virus production. Cell Host Microbe 23 (6):819–831 e815. doi:10.1016/j.chom.2018.05.005 10.1016/j.chom.2018.05.005 29902443

[146] Leier HC, Weinstein JB, Kyle JE, Lee JY, Bramer LM, Stratton KG, Kempthorne D, Navratil AR, Tafesse EG, Hornemann T, Messer WB, Dennis EA, Metz TO, Barklis E, Tafesse FG (2020). A global lipid map defines a network essential for Zika virus replication. Nat Commun.

[147] McMahon HT, Gallop JL (2005). Membrane curvature and mechanisms of dynamic cell membrane remodelling. Nature.

[148] Liebscher S, Ambrose RL, Aktepe TE, Mikulasova A, Prier JE, Gillespie LK, Lopez-Denman AJ, Rupasinghe TWT, Tull D, McConville MJ, Mackenzie JM (2018). Phospholipase A2 activity during the replication cycle of the flavivirus West Nile virus. PLoS Pathog.

[149] Hsu NY, Ilnytska O, Belov G, Santiana M, Chen YH, Takvorian PM, Pau C, van der Schaar H, Kaushik-Basu N, Balla T, Cameron CE, Ehrenfeld E, van Kuppeveld FJ, Altan-Bonnet N (2010). Viral reorganization of the secretory pathway generates distinct organelles for RNA replication. Cell.

[150] Aktepe TE, Pham H, Mackenzie JM (2015). Differential utilisation of ceramide during replication of the flaviviruses West Nile and dengue virus. Virology.

